# Association of possible sarcopenia and its components with all-cause mortality in a middle-aged and older population: a 9-year cohort study

**DOI:** 10.1038/s41598-025-16034-0

**Published:** 2025-08-20

**Authors:** Zhiping Duan, Guihua Jiang, Yingxia Zhu, Wei Huang, Yunda Huang, Hong Yang

**Affiliations:** Department of Geriatrics, The Third People’s Hospital of Yunnan Province, The Second Affiliated Hospital of Dali University, 292 Beijing Road, Kunming, 650011 Yunnan Province China

**Keywords:** Possible sarcopenia, Handgrip strength, 5-time chair stand test, All-cause mortality, CHARLS, Geriatrics, Nutrition

## Abstract

The association of possible sarcopenia and its components with all-cause mortality needs further study. This study analyzed the association of possible sarcopenia, handgrip strength and 5-time chair stand test with all-cause mortality in a Chinese middle-aged and older community population. This cohort study included a total of 11,233 participants aged ≥ 45 years from the 2011–2020 China Health and Retirement Longitudinal Study. Diagnostic criteria for possible sarcopenia are based on Asian Working Group for Sarcopenia 2019: low handgrip strength or 5-time chair stand test ≥ 12 s. The association of possible sarcopenia and its components with all-cause mortality was assessed by Cox proportional risk models. The results showed that possible sarcopenia was associated with an increased risk of all-cause mortality, hazard ratio (HR) and 95% confidence interval (CI) 1.77 (1.55–2.02), *p* < 0.001. Both low handgrip strength and 5-time chair stand test ≥ 12 s significantly increased the risk of all-cause mortality, with HRs and 95%CIs of 1.51 (1.18–1.93), *P* = 0.001 and 1.61 (1.39–1.87), *P* < 0.001, respectively; when both were present, the risk of all-cause mortality was further elevated, HR and 95%CI 2.54 (2.11–3.06), *P* < 0.001. Possible sarcopenia and its components are all associated with a higher mortality and it is important to identify these risk factors in primary care.

## Introduction

Sarcopenia is a degenerative syndrome primarily associated with aging, with a prevalence of 5.5% to 25.7% in the older Asian population and is characterized by low muscle mass with low muscle strength or physical performance decline^1,2^. Muscle mass peaks at 20 years of age and gradually decreases after 30 years of age, and between 40 and 70 years of age, muscle mass decreases by 8% per decade; after 70 years of age, it decreases by nearly 10% in 10 years. Muscle strength decreases more rapidly than muscle mass, by 25–35% in 10 years after 70 years of age^3^. Physical performance tends to decrease as muscle mass and muscle strength decline, making sarcopenia more common in the older population. Older adults with sarcopenia are limited in their daily activities, leading to an increased risk of weakness, falls, fractures, and disability, which in turn increases the risk of hospitalization and death^4–7^. Studies from several countries have shown that sarcopenia is associated with a 41%-114% increased risk of death in community-based adults^8–11^ and hospitalized patients^12^. This not only severely impairs the quality of life of the older but also places a heavy burden on the healthcare system^13^. Existing diagnostic criteria for sarcopenia require a comprehensive assessment of muscle mass, muscle strength, and muscle function, which are often time-consuming, labor-intensive, and impractical for large population-based surveys. European Working Group on Sarcopenia in Older People 2 (EWGSOP2)^14^ and Asian Working Group for Sarcopenia 2019 (AWGS2019)^1^ proposed a new concept of possible sarcopenia, which is characterized by a decrease in muscle strength or physical performance, assessed by handgrip strength and the 5-time chair stand test. The indicators and procedures used to identify possible sarcopenia are much simpler and less expensive than those needed to fully diagnose sarcopenia^1,14^. In primary care clinics and communities, this convenient method facilitates early screening for sarcopenia, leading to better management of the risk of sarcopenia^15^.

As the population ages, it has become imperative to address the health impact of sarcopenia. That’s why the concept of possible sarcopenia^1,14^ was introduced in the latest consensus to enable timely intervention in the absence of the technology needed to fully diagnose sarcopenia. It has been demonstrated that possible sarcopenia increases the incidence of depression^16^, chronic kidney disease^17^, and type 2 diabetes mellitus^18^ and increases the risk of all-cause mortality in patients with cancer^19^. The dangers of sarcopenia have been studied in some depth, but the health effects of possible sarcopenia have not been adequately researched, which is clearly not conducive to drawing attention to this new concept.

There is a lack of research data on the risk of all-cause mortality in a community-based population in Asia with possible sarcopenia. We conducted a 9-year cohort study to estimate the association between possible sarcopenia and its components with all-cause mortality in a community-based middle-aged and older population in China, using nationally representative data from the China Health and Retirement Longitudinal Study (CHARLS). By assessing possible sarcopenia and analyzing its health effects using easily accessible indicators, this study not only provides a convenient tool for primary care to identify health risks, but also draws attention to the pre-morbid stages of sarcopenia and helps to prevent it.

## Methods

### Study population

**Fig. 1:**
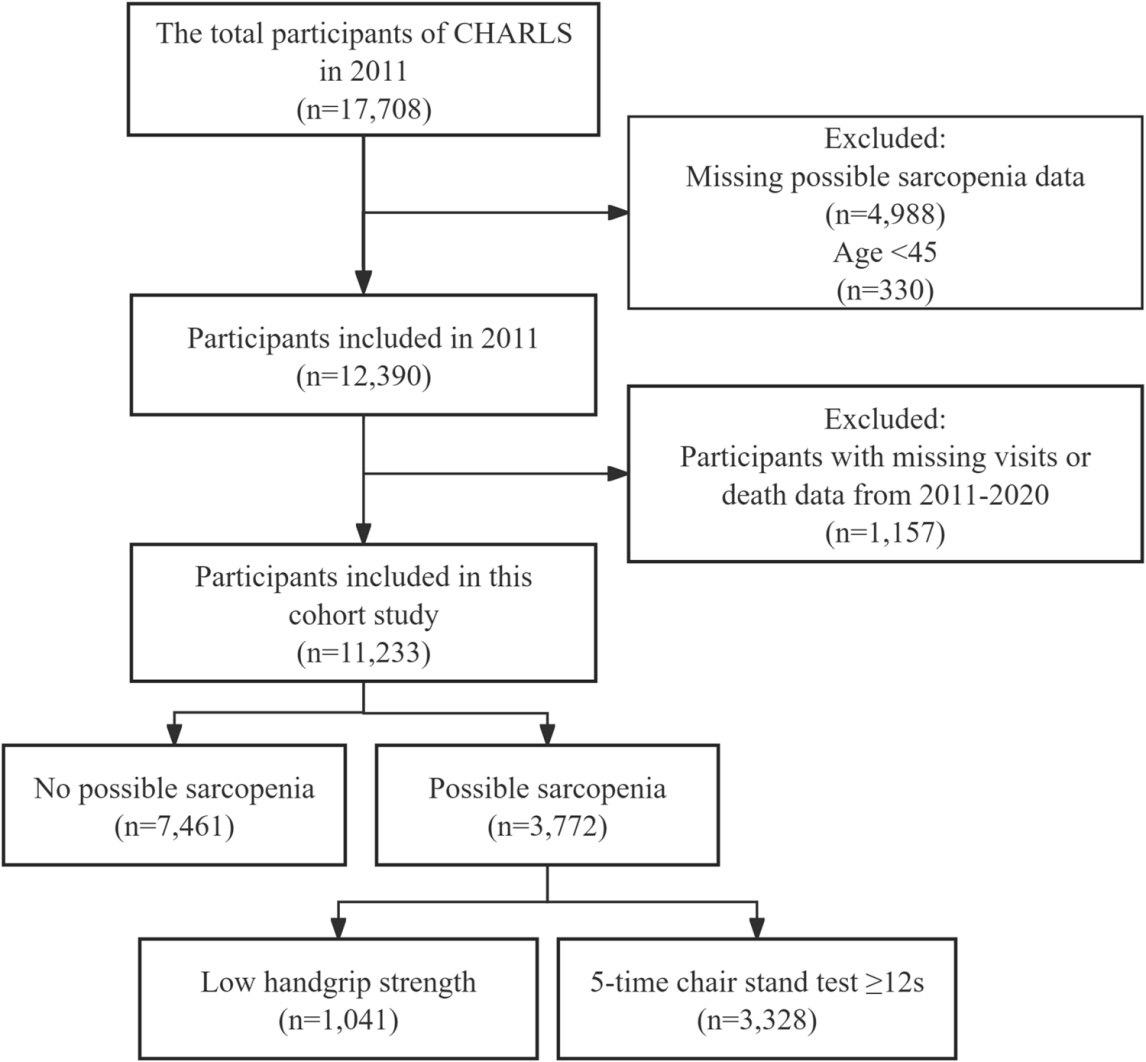
Flow chart of the screening of participants.

Our study was drawn from the China Health and Retirement Longitudinal Study (CHARLS), a national longitudinal study focused on community-dwelling middle-aged and older adults in China. The baseline survey was conducted in 2011, followed by follow-up visits every 2–3 years. The CHARLS survey data contains information on a wide range of topics, from personal health to socioeconomics, and its detailed information and methodology have been reported in previous articles^20^. Specifically, our cohort study included participants from the baseline survey of CHARLS in 2011, with exclusion criteria: (1) missing data on possible sarcopenia in 2011 (n = 4988), (2) age < 45 years (n = 330), and (3) missing data on loss to follow-up or deaths at follow-up in 2013, 2015, 2018, and 2020 (n = 1157). Ultimately, 11,233 participants with complete information were included (Fig. 1).

CHARLS informed consent was obtained from each participant before conducting the investigation. The CHARLS study was approved by the Biomedical Ethics Committee of Peking University (approval number: IRB00001052-11015)^20^. The study was conducted in accordance with the Strengthening the Reporting of Observational Studies in Epidemiology (STROBE) guideline recommendations related to cohort studies^21^.

### Evaluation of possible sarcopenia status

The AWGS2019 proposes a definition of possible sarcopenia: participant has low muscle strength or low physical performance^1^. Muscle strength was assessed based on handgrip strength, which was measured twice for each hand using a YuejianTM WL-1000 dynamometer manufactured by Nantong Yuejian Physical Testing Equipment Co. Participants were asked to stand and grasp the grip strength meter with their elbows at a right angle and hold it firmly for a few seconds before releasing it. Low muscle strength was defined as maximum handgrip strength < 28 kg for men and < 18 kg for women. Physical performance was assessed by performing 5-time chair stand test using a 47-cm high stool and stopwatch. Participants were asked to cross their arms over their chests, stand up straight as fast as they could and then sit down for five repetitions, without stopping between each repetition, and without pushing with their arms. Participants were considered to have low physical performance when their 5-time chair stand test were ≥ 12 s. Based on the above diagnostic criteria, we assessed the possible sarcopenia status of the participants included in 2011.

### Mortality data

Mortality data were obtained from questionnaires conducted in 2013, 2015, 2018, and 2020, with a follow-up period of approximately 9 years. The interviewer visited the participant’s residence, and, in the event of the participant’s death, information was gathered by interviewing family members who lived with the deceased.

### Covariates

In this cohort study, covariates included information on participants’ demographics, lifestyle habits, and chronic disease status. Information on these covariates was obtained from the 2011 baseline questionnaire in CHARLS. Demographic information included gender, age, education, marital, and residence. Lifestyle habits included smoking and alcohol consumption. Chronic disease status included hypertension, dyslipidemia, diabetes, cancer, chronic lung diseases, liver disease, heart disease, stroke, kidney disease, digestive disease, arthritis, and asthma, and this information was based on the question “Have you been diagnosed with a chronic disease by a doctor?”.

### Statistical analysis

Continuous variables are expressed as mean and standard deviation (SD), and categorical variables are expressed as counts and percentages. Participants’ baseline characteristics in 2011 were compared using chi-square test and t-test. Hazard ratios (HRs) and 95% confidence intervals (CIs) for possible sarcopenia, handgrip strength, and 5-time chair stand test to mortality were estimated using the Cox proportional hazards model. We developed 3 models: model 1 was a univariate analysis; model 2 adjusted for demographic information and lifestyle habits: gender, age, education, marital, residence, smoke, and drink; and model 3 further adjusted for chronic disease states based on model 2: hypertension, dyslipidemia, diabetes, cancer, chronic lung diseases, liver disease, heart disease, stroke, kidney disease, digestive disease, arthritis, and asthma. A Kaplan-Meyer survival curve was used to analyze how the probability of survival changed over time for participants with possible sarcopenia, low handgrip strength, and 5-time chair stand test ≥ 12 s. The linear relationship between handgrip strength and 5-time chair stand test with mortality was further validated using restricted cubic spline (RCS) models.

In order to verify the stability of the results, we conducted 3 sensitivity analyses: (1) We analyzed subgroups of participants of different genders, ages, hypertension, diabetes, chronic lung diseases, heart disease, and kidney disease to test the stability of the results in different subgroups. (2) Considering the effect of stroke on handgrip strength and the 5-time chair stand test, we reran the Cox regression after excluding those with stroke at baseline. (3) To further reduce the effect of confounders, we performed propensity score matching, with a caliper value set at 0.2 and using 1:1 nearest-neighbor matching, to analyze the associations between possible sarcopenia, low handgrip strength, and 5-time chair stand test ≥ 12 s with all-cause mortality.

All the analyses were performed with the statistical software Stata/MP version 17.0 (StataCorp LP, College Station, TX, USA), Free Statistics software version 1.9.2 (Beijing Free Clinical Medical Technology Co., Ltd.), and R software version 4.3.0. The level of statistical significance was set at *p* < 0.05 (two-sided).

## Results

### Baseline characteristics of study participants

Baseline characteristics of the participants grouped based on the presence or absence of possible sarcopenia are shown in Table 1. A total of 11,233 participants, age 59.3 ± 9.3 and 51.5% females, were included in this study. Of these, 3772 were diagnosed with possible sarcopenia. Compared to participants with no possible sarcopenia, participants with possible sarcopenia were more likely to be female, older, less educated, live in rural areas, more alcohol consumption, be more likely to have hypertension, diabetes, chronic lung disease, heart disease, stroke, kidney disease, arthritis, and asthma (all *P* < 0.05).

**Table 1:** Baseline characteristics of participants.

	Total (n = 11,233)	No possible sarcopenia (n = 7461)	Possible sarcopenia (n = 3772)	*P*-value
Gender, n (%)				< 0.001
Female	5,782 (51.5)	3599 (48.2)	2183 (57.9)	
Male	5,451 (48.5)	3862 (51.8)	1589 (42.1)	
Age, year, mean ± SD	59.3 ± 9.3	57.3 ± 8.2	63.1 ± 10.0	< 0.001
Education, n (%)				< 0.001
Primary school or below	7,822 (69.6)	4778 (64)	3,044 (80.7)	
Middle school	2,276 (20.3)	1748 (23.4)	528 (14)	
High school or above	1,135 (10.1)	935 (12.5)	200 (5.3)	
Marital, n (%)				< 0.001
Other	1,322 (11.8)	653 (8.8)	669 (17.7)	
Married	9,911 (88.2)	6808 (91.2)	3103 (82.3)	
Residence, n (%)				< 0.001
Urban areas	1,847 (16.5)	1340 (18)	507 (13.5)	
Rural areas	9,375 (83.5)	6113 (82)	3262 (86.5)	
Smoking, n (%)				< 0.001
Never smoked	6,679 (59.7)	4309 (57.9)	2370 (63.3)	
Former smoker	971 ( 8.7)	640 (8.6)	331 (8.8)	
Current smoker	3,534 (31.6)	2490 (33.5)	1044 (27.9)	
Drinking, n (%)				< 0.001
Never or rarely	7,440 (66.3)	4688 (62.9)	2752 (73)	
Less than once a month	904 ( 8.1)	669 (9)	235 (6.2)	
More than once a month	2,882 (25.7)	2099 (28.2)	783 (20.8)	
Chronic disease status				
Hypertension, n (%)	2,619 (23.5)	1526 (20.6)	1093 (29.2)	< 0.001
Dyslipidemia, n (%)	942 ( 8.6)	620 (8.5)	322 (8.7)	0.628
Diabetes, n (%)	597 ( 5.4)	372 (5)	225 (6)	0.028
Cancer, n (%)	108 ( 1.0)	68 (0.9)	40 (1.1)	0.434
Chronic lung diseases, n (%)	1,151 (10.3)	681 (9.2)	470 (12.5)	< 0.001
Liver disease, n (%)	437 ( 3.9)	277 (3.7)	160 (4.3)	0.168
Heart disease, n (%)	1,217 (10.9)	673 (9.1)	544 (14.5)	< 0.001
Stroke, n (%)	206 ( 1.8)	86 (1.2)	120 (3.2)	< 0.001
Kidney disease, n (%)	693 ( 6.2)	425 (5.7)	268 (7.2)	0.003
Digestive disease, n (%)	2,602 (23.3)	1695 (22.8)	907 (24.1)	0.115
Arthritis, n (%)	3,811 (34.0)	2402 (32.3)	1409 (37.4)	< 0.001
Asthma, n (%)	415 ( 3.7)	231 (3.1)	184 (4.9)	< 0.001
Biomarkers				
Handgrip strength, kg, mean ± SD	32.9 ± 10.2	35.4 ± 9.4	27.9 ± 10.0	< 0.001
5-time chair stand test, s, mean ± SD	10.8 ± 4.4	8.8 ± 1.8	14.9 ± 5.1	< 0.001

SD, standard deviation.

### Association between possible sarcopenia with all-cause mortality.

**Fig. 2:**
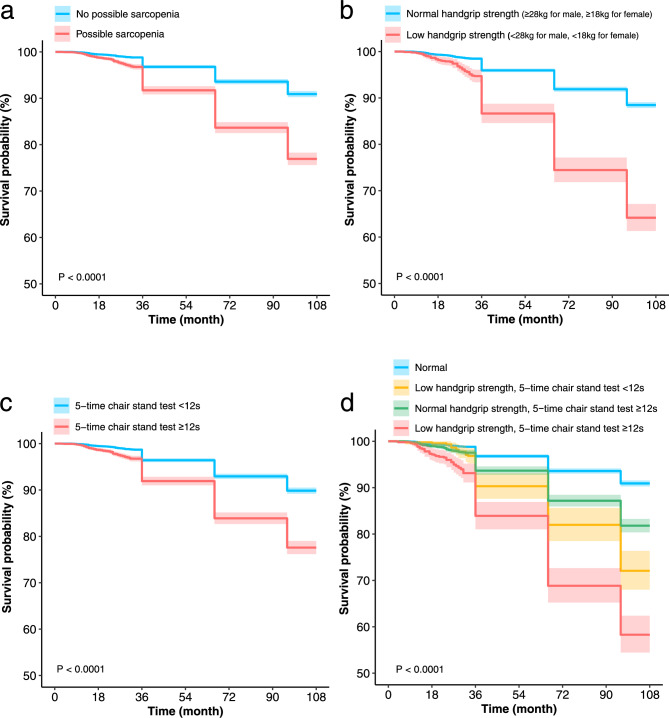
Kaplan–Meier survival curves associated with risk of all-cause mortality in participants with possible sarcopenia (**a**), low handgrip strength (**b**), 5-time chair stand test ≥ 12 s (**c**), and combinations of components (**d**).

After 9 years of follow-up, there were 1,548 deaths. In model 1 of the univariate analysis, all-cause mortality was significantly higher in possible sarcopenia compared with no possible sarcopenia, HR and 95% CI 2.75 (2.48–3.04), *P* < 0.001. In model 2 adjusted for gender, age, education, marriage, residence, smoking, and drinking, HR and 95% CI 1.77 (1.56–2.02), *P* < 0.001. In fully adjusted model 3, HR and 95% CI 1.77 (1.56–2.02), *P* < 0.001 (Table 2). In the Kaplan–Meier survival curve, participants with possible sarcopenia had a higher risk of all-cause mortality over time, *P* < 0.0001 for the log-rank test (Fig. 2a).

**Table 2:** Association between possible sarcopenia, handgrip strength, and 5-time chair stand test with all-cause mortality.

	Cases (%)	Model 1	*P*-value		Model 2	*P*-value		Model 3	*P*-value
		HR (95% CI)			HR (95% CI)			HR (95% CI)	
No possible sarcopenia	678 (9.1)	1(Ref)			1(Ref)			1(Ref)	
Possible sarcopenia	870 (23.1)	2.75 (2.48–3.04)	< 0.001		1.77 (1.56–2.02)	< 0.001		1.77 (1.55–2.02)	< 0.001
Handgrip strength									
Continuous variable									
Handgrip strength (per 1 kg)	1548 (13.8)	0.97 (0.96–0.97)	< 0.001		0.97 (0.96–0.97)	< 0.001		0.97 (0.96–0.98)	< 0.001
Categorical variable									
Normal handgrip strength (≥ 28 kg for male, ≥ 18 kg for female)	1175 (11.5)	1(Ref)			1(Ref)			1(Ref)	
Low handgrip strength (< 28 kg for male, < 18 kg for female)	373 (35.8)	3.6 (3.2–4.04)	< 0.001		1.67 (1.45–1.93)	< 0.001		1.67 (1.45–1.93)	< 0.001
5-time chair stand test									
Continuous variable									
5-time chair stand test (per 1 s)	1548 (13.8)	1.05 (1.04–1.05)	< 0.001		1.03 (1.02–1.03)	< 0.001		1.02 (1.02–1.03)	< 0.001
Categorical variable									
5-time chair stand test < 12 s	802 (10.1)	1(Ref)			1(Ref)			1(Ref)	
5-time chair stand test ≥ 12 s	746 (22.4)	2.37 (2.15–2.62)	< 0.001		1.72 (1.51–1.95)	< 0.001		1.7 (1.5–1.94)	< 0.001
Combination of components									
Normal	678 (9.1)	1(Ref)			1(Ref)			1(Ref)	
Low handgrip strength, 5-time chair stand test < 12 s	124 (27.9)	3.39 (2.8–4.11)	< 0.001		1.5 (1.17–1.91)	0.001		1.51 (1.18–1.93)	0.001
Normal handgrip strength, 5-time chair stand test ≥ 12 s	497 (18.2)	2.11 (1.88–2.36)	< 0.001		1.62 (1.4–1.87)	< 0.001		1.61 (1.39–1.87)	< 0.001
Low handgrip strength, 5 − time chair stand test ≥ 12 s	249 (41.7)	5.66 (4.89–6.54)	< 0.001		2.58 (2.15–3.09)	< 0.001		2.54 (2.11–3.06)	< 0.001

Model 1: unadjusted.

Model 2: adjusted for gender, age, education, marital, residence, smoking, and drinking.

Model 3: further adjusted hypertension, dyslipidemia, diabetes, cancer, chronic lung diseases, liver disease, heart disease, stroke, kidney disease, digestive disease, arthritis and asthma.

HR, hazards ratio; CI, confidence interval.

### Association between handgrip strength and 5-time chair stand test with all-cause mortality.

**Fig. 3:**
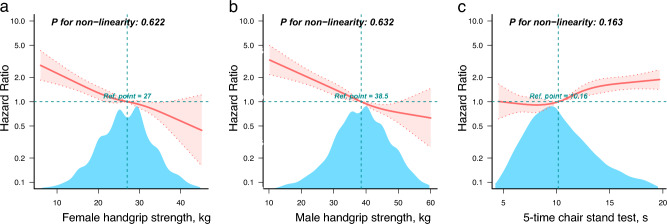
RCS model of the association of handgrip strength and 5-time chair stand test with all-cause mortality. **a** and **b** Denote the RCS models of handgrip strength for females and males, respectively, included only 99% of participants, had knots of 4. **c** Denotes the RCS model for the 5-time chair stand test, included only 96% of participants, had knots of 5. Adjusted for all covariates: gender, age, education, marital, residence, smoking, drinking, hypertension, dyslipidemia, diabetes, cancer, chronic lung diseases, liver disease, heart disease, stroke, kidney disease, digestive disease, arthritis, and asthma. The red solid and dashed lines represent HR and 95CI, respectively. HR, hazards ratio; CI, confidence interval. RCS, restricted cubic spline.

When handgrip strength was used as a continuous variable, there was a 3% decrease in mortality when handgrip strength was increased by every 1 kg in all three models, all *P* < 0.001 (Table 2). Considering that there were significant gender differences in handgrip strength, we stratified the analysis according to gender in the RCS model. Figure 3a,b show the results of the RCS model for handgrip strength and mortality for females and males, and no non-linear association, both *P* for nonlinearity test > 0.05. When handgrip strength was used as a categorical variable, the HRs and 95% CIs for low handgrip strength were 3.6 (3.2–4.04), 1.67 (1.45–1.93), and 1.67 (1.45–1.93) in models 1, 2, and 3, respectively, all *P* < 0.001 (Table 2). In the Kaplan–Meier survival curve, survival was lower in the low handgrip strength group, log-rank test *P* < 0.0001 (Fig. 2b).

When the 5-time chair stand test was used as a continuous variable, each second increase in time increased mortality by 5%, 3%, and 2% in models 1, 2, and 3, respectively, all *P* < 0.001 (Table 2). Figure 3c shows the results of the 5-time chair stand test with RCS model for mortality; no nonlinear association was found, P for the nonlinearity test was 0.163. When 5-time chair stand test was used as a categorical variable, the HRs and 95% CIs for 5-time chair stand test ≥ 12 s in models 1, 2, and 3 were 2.37 (2.15–2.62), 1.72 (1.51–1.95), and 1.7 (1.5–1.94), respectively, all *P* < 0.001 (Table 2). In the Kaplan–Meier survival curve, the 5-time chair stand test ≥ 12 s group had a lower survival rate, log-rank test *P* < 0.0001 (Fig. 2c).

We further analyzed the separate and combined effects of low handgrip strength and 5-time chair stand test ≥ 12 s on mortality. When only low handgrip strength was present, the risk of death increased by 51%, HR and 95%CI 1.51 (1.18–1.93), *P* = 0.001; when only the 5-time chair stand test ≥ 12 s was present, the risk of death increased by 61%, HR and 95%CI 1.61 (1.39–1.87), *P* < 0.001; and when both were present, the risk of death increased by 154%, HR and 95%CI 2.54 (2.11–3.06), *P* < 0.001 (Table 2). In the Kaplan–Meier survival curve, survival was lowest when both were present, log-rank test *P* < 0.0001 (Fig. 2d).

### Sensitivity analysis

**Fig. 4:**
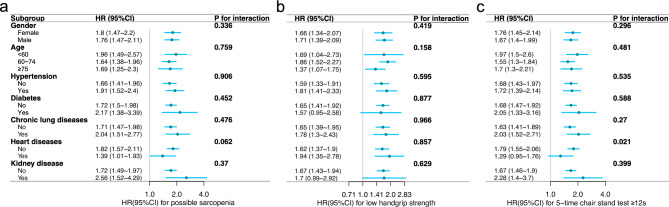
Forest plot of subgroup analysis of the association between possible sarcopenia (**a**), low handgrip strength (**b**), and 5-time chair stand test ≥ 12 s (**c**) with all-cause mortality. Blue squares and lines represent HR and 95% CI, respectively. Adjusted for all covariates: gender, age, education, marital, residence, smoking, drinking, hypertension, dyslipidemia, diabetes, cancer, chronic lung diseases, lver diseaseand, heart disease, stroke, kidney disease, digestive disease, arthritis, and asthma. HR, hazards ratio; CI, confidence interval.

We performed subgroup analyses based on gender, age (< 60, 60–74, ≥ 75), hypertension, diabetes, chronic lung disease, heart diseases, and kidney disease to further determine the relationship between possible sarcopenia, low handgrip strength, and 5-time chair stand test ≥ 12 s with all-cause mortality. After adjusted for all covariates, the associations between possible sarcopenia and low handgrip strength with mortality remained consistent across all subgroups (Fig. 4a,b). There was a difference in the association between the 5-time chair stand test and mortality in the heart disease subgroup, P for interaction = 0.021 (Fig. 4c).

After excluded participants with stroke at baseline, we reran univariate and multifactorial Cox modeling analyses, and the associations of possible sarcopenia and its components with all-cause mortality were similar to previous results (Supplementary Table 1).

Supplementary Table 2 shows the results of the propensity score matching. Supplementary Figs. 1–3 show the standardized mean difference (SMD) for all covariates; there were no significant differences in baseline characteristics between groups. Results after propensity score matching were similar to previous ones, with possible sarcopenia, low handgrip strength, and 5-time chair stand test ≥ 12 s all significantly associated with all-cause mortality.

## Discussion

The CHARLS, with participants from different geographic and economic regions of China, is able to represent the general state of China’s middle-aged and older community-based population. In this cohort study, we used the criteria of the AWGS2019 to define possible sarcopenia. After adjusting for all covariates, possible sarcopenia was significantly associated with a 77% increase in all-cause mortality. Further analysis showed that for every 1 kg increase in handgrip strength, there was a 3% decrease in mortality; low handgrip strength resulted in a 51% increase in mortality. Mortality increased by 2% for each second extension of the 5-time chair stand test; when the 5-time chair stand test ≥ 12 s, the risk of mortality increased by 61%. When both components of possible sarcopenia were present, the risk of mortality was significantly increased by 154%. To reduce selection bias, we used PSM and IPTW for sensitivity analysis and got similar results to the previous analysis.

Our study shows that the prevalence of possible sarcopenia in the middle-aged and older population was very prevalent at 33.6%, which is higher than the overall prevalence of possible sarcopenia in Chinese people over 40 years of age, which was 23.6% as indicated in a previous study by Zhao et al.^22^. This may be because we included people ≥ 45 years of age, and the prevalence of possible sarcopenia increases with age^22^. We also found that the prevalence of possible sarcopenia was significantly greater in females than in males (57.9% vs. 42.1%). Previous findings have confirmed the existence of gender differences in low somatic functioning, suggesting that females are more susceptible than males to possible sarcopenia^23^.

The adverse effects of sarcopenia have been demonstrated, but studies for possible sarcopenia are insufficient. Sarcopenia is associated with an increased risk of death in patients with non-alcoholic fatty liver disease^24^, type 2 diabetes mellitus^25^, and chronic kidney disease^26^. There are also meta-analyses and studies confirming that sarcopenia increases the mortality rate in community-based populations in various countries^10,11,27–29^, inpatient hospitalizations^30,31^, and nursing home residents^32,33^. Therefore, sarcopenia should require early screening, diagnosis, and intervention, just like other chronic diseases such as hypertension, diabetes, and dyslipidemia. However, the diagnosis of sarcopenia is currently not only dependent on sophisticated equipment such as magnetic resonance imaging, computed tomography, dual-energy X-ray absorptiometry, or bioelectrical impedance analysis, but also requires a careful assessment of physical performance. Obviously, the diagnosis of sarcopenia is more costly than that of other common chronic diseases, which is not conducive to the promotion of early management of sarcopenia.

In order to facilitate the early identification of sarcopenia, both EWGSOP2 and AWGS2019 have proposed a new concept of possible sarcopenia based on low muscle strength and low physical performance. This simple definition requires only the use of convenient and inexpensive devices such as grip strength meters, chairs, and stopwatches for diagnosis, making it highly feasible for people to be assessed in primary care settings or even in their homes. Currently, there is insufficient research devoted to possible sarcopenia. Although it has been demonstrated that possible sarcopenia increases the incidence of depression^16^, chronic kidney disease^17^, and type 2 diabetes mellitus^18^ and increases the risk of all-cause mortality in patients with cancer^19^, its association with all-cause mortality in community-based populations is controversial. In a study of 4,612 United States, Finnish, and Dutch community-based populations^34^, possible sarcopenia based on EWGSOP2 was associated with a 61% increased risk of all-cause mortality. In another study that included 103 Turkish community-based populations^35^, EWGSOP2-based possible sarcopenia was not significantly associated with all-cause mortality. To the best of our knowledge, there is a lack of studies on the association between possible sarcopenia based on AWGS2019 and mortality in Asian community-based populations, so our study fills this gap and helps primary care workers and community residents to pay more attention to the relatively new concept of possible sarcopenia. In addition, our findings that both low grip strength and a longer 5-time chair stand test time were associated with higher mortality, and that the risk of mortality was further increased when both were present, provide further evidence of the harm caused by possible sarcopenia.

In subgroup analyses, the associations between possible sarcopenia and low handgrip strength with mortality did not differ in all subgroups. In the heart disease subgroup, the association between 5 chair stand tests ≥ 12 and mortality was different, and this association was more pronounced in participants without heart disease. This may be due to the fact that the 5-time chair stand test involves performing the sit-down and get-up maneuvers as fast as one is able, and participants with heart disease may have been concerned that their condition would worsen and thus affect their performance, leading to biased results.

The impact of possible sarcopenia on mortality risk comes from the negative effects of low muscle strength and low physical performance. First, individuals with decreased muscle strength and physical performance have an increased risk of falls and fractures, and these can lead to mobility problems, muscle atrophy, and infections, which increase the risk of death^36,37^. Second, decreased muscle strength and physical performance are associated with malnutrition^38,39^, and malnutrition exacerbates the risk of death in people with a variety of chronic illnesses^40,41^. And lastly, the presence of a number of physiologic, immune, and metabolic function changes, such as inflammation, metabolic abnormalities, and oxidative stress, are key factors linking sarcopenia to mortality^42^; the association between possible sarcopenia and these factors needs to be confirmed by further studies.

In conclusion, sarcopenia is a muscular aging disease that is associated with a variety of adverse outcomes, and the concept of possible sarcopenia provides an early warning signal for this aging. The significance of our study is that it confirms that possible sarcopenia should not only be used as an early warning sign, but also that the adverse effects of this signal itself should be emphasized, as it is strongly associated with all-cause mortality in a middle-aged and older community population. To the best of our knowledge, this is the first study to specifically address the association between possible sarcopenia and its components with all-cause mortality in a Chinese middle-aged and older community-based population. Although some previous studies have separately analyzed the relationship between low hand grip strength and poor physical functioning with mortality, there are no studies that have combined the two as a new label for possible sarcopenia and analyzed their combined effect on mortality. ‘Probable sarcopenia’ as defined by the AWGS2019 criteria provides a clinically actionable threshold for early identification. This standardized definition facilitates risk stratification in the primary care setting, especially in the absence of advanced diagnostic tools such as DXA. Our findings confirm that this simplified diagnostic method that combines handgrip strength and physical function is equally good at capturing mortality risk, justifying its use as a screening method. We used a representative sample, which makes the findings well generalizable. Also, we used different statistical methods for analysis to verify the stability of the findings. However, there are some limitations to our study. Firstly, due to the lack of detailed cause-of-death data, we were unable to accurately assess the precise causes of death associated with possible sarcopenia. Second, our diagnostic criteria were derived from AWGS2019, and their applicability to other diagnostic criteria and other national populations needs to be confirmed by further research. Third, with a high proportion of rural residents in our study (83.5%), differences in health care access, health awareness, and lifestyle between urban and rural areas may affect the results of the study. Therefore, caution is needed in generalizing these findings to urban populations. In addition, further interventional studies are needed in the future to examine the impact of interventions for possible sarcopenia on reducing all-cause mortality.

## Conclusion

Possible sarcopenia, low handgrip strength and longer 5-time chair stand test time are all associated with higher mortality and it is important to identify these risk factors in primary care.

## Supplementary Information

Supplementary Information.

## Data Availability

The data used in our study comes from China Health and Retirement Longitudinal Study (CHARLS), a publicly available database. This data can be found here: https://charls.pku.edu.cn/.
