# Supplementary material for: Association of possible sarcopenia and its components with all-cause mortality in a middle-aged and older population: a 9-year cohort study
Source: Sci Rep. 2025 Aug 20;15:30481. doi: 10.1038/s41598-025-16034-0 (PMC12365224; doi:10.1038/s41598-025-16034-0)
Supplement: Supplementary file 1 — Supplementary Information. [file 41598_2025_16034_MOESM1_ESM.docx]

**Supplementary Table 1. Association between possible sarcopenia, handgrip strength, and 5-time chair stand test with all-cause mortality after excluded participants with strokes.**

|  | **Cases (%)** | **Model 1** |  |  | **Model 2** |  |  | **Model 3** |  |
| --- | --- | --- | --- | --- | --- | --- | --- | --- | --- |
|  |  | **HR (95 % CI)** | **P-value** |  | **HR (95 % CI)** | **P-value** |  | **HR (95 % CI)** | **P-value** |
| **No possible sarcopenia** | 665 (9) | 1(Ref) |  |  | 1(Ref) |  |  | 1(Ref) |  |
| **Possible sarcopenia** | 821 (22.5) | 2.69 (2.43~2.98) | <0.001 |  | 1.72 (1.5~1.96) | <0.001 |  | 1.71 (1.5~1.96) | <0.001 |
| **Handgrip strength** |  |  |  |  |  |  |  |  |  |
| **Continuous variable** |  |  |  |  |  |  |  |  |  |
| Handgrip strength (per 1 kg) | 1486 (13.5) | 0.97 (0.96~0.97) | <0.001 |  | 0.97 (0.96~0.98) | <0.001 |  | 0.97 (0.96~0.98) | <0.001 |
| **Categorical variable** |  |  |  |  |  |  |  |  |  |
| Normal handgrip strength (≥28kg for male, ≥18kg for female) | 1134 (11.4) | 1(Ref) |  |  | 1(Ref) |  |  | 1(Ref) |  |
| Low handgrip strength (<28kg for male, <18kg for female) | 352 (35.1) | 3.57 (3.16~4.02) | <0.001 |  | 1.62 (1.39~1.88) | <0.001 |  | 1.63 (1.4~1.89) | <0.001 |
| **5-time chair stand test** |  |  |  |  |  |  |  |  |  |
| **Continuous variable** |  |  |  |  |  |  |  |  |  |
| 5-time chair stand test (per 1 s) | 1486 (13.5) | 1.05 (1.04~1.05) | <0.001 |  | 1.02 (1.02~1.03) | <0.001 |  | 1.02 (1.01~1.03) | <0.001 |
| **Categorical variable** |  |  |  |  |  |  |  |  |  |
| 5-time chair stand test <12s | 787 (10.1) | 1(Ref) |  |  | 1(Ref) |  |  | 1(Ref) |  |
| 5-time chair stand test ≥12s | 699 (21.8) | 2.31 (2.09~2.56) | <0.001 |  | 1.67 (1.47~1.9) | <0.001 |  | 1.66 (1.46~1.89) | <0.001 |
| **Combination of components** |  |  |  |  |  |  |  |  |  |
| Normal | 665 (9) | 1(Ref) |  |  | 1(Ref) |  |  | 1(Ref) |  |
| Low handgrip strength, 5−time chair stand test <12s | 122 (27.8) | 3.39 (2.8~4.11) | <0.001 |  | 1.45 (1.14~1.85) | 0.003 |  | 1.46 (1.14~1.87) | 0.003 |
| Normal handgrip strength, 5−time chair stand test ≥12s | 469 (17.8) | 2.06 (1.83~2.32) | <0.001 |  | 1.58 (1.36~1.83) | <0.001 |  | 1.57 (1.35~1.82) | <0.001 |
| Low handgrip strength, 5−time chair stand test ≥12s | 230 (40.8) | 5.52 (4.75~6.42) | <0.001 |  | 2.45 (2.03~2.95) | <0.001 |  | 2.45 (2.03~2.96) | <0.001 |

Model 1: unadjusted.

Model 2: adjusted for gender, age, education, marital, residence, smoking, and drinking.

Model 3: further adjusted hypertension, dyslipidemia, diabetes, cancer, chronic lung diseases, liver disease, heart disease, kidney disease, digestive disease, arthritis and asthma.

Abbreviations: HR, hazards ratio; CI, confidence interval.

**Supplementary Table 2. Results for propensity scoring.**

|  | **Unmatched.crude** |  |  | **PropensityScore.Matched** |  |
| --- | --- | --- | --- | --- | --- |
|  | **HR (95 % CI)** | **P-value** |  | **HR (95 % CI)** | **P-value** |
| **Possible sarcopenia** | 3 (2.65~3.39) | <0.001 |  | 1.59 (1.37~1.84) | <0.001 |
| **Low handgrip strength** | 3.76 (3.28~4.31) | <0.001 |  | 1.55 (1.29~1.86) | <0.001 |
| **5-time chair stand test ≥12s** | 2.6 (2.3~2.93) | <0.001 |  | 1.59 (1.37~1.83) | <0.001 |

Abbreviations: IPTW, inverse probability of treatment weighting; HR, hazards ratio; CI, confidence interval.

**
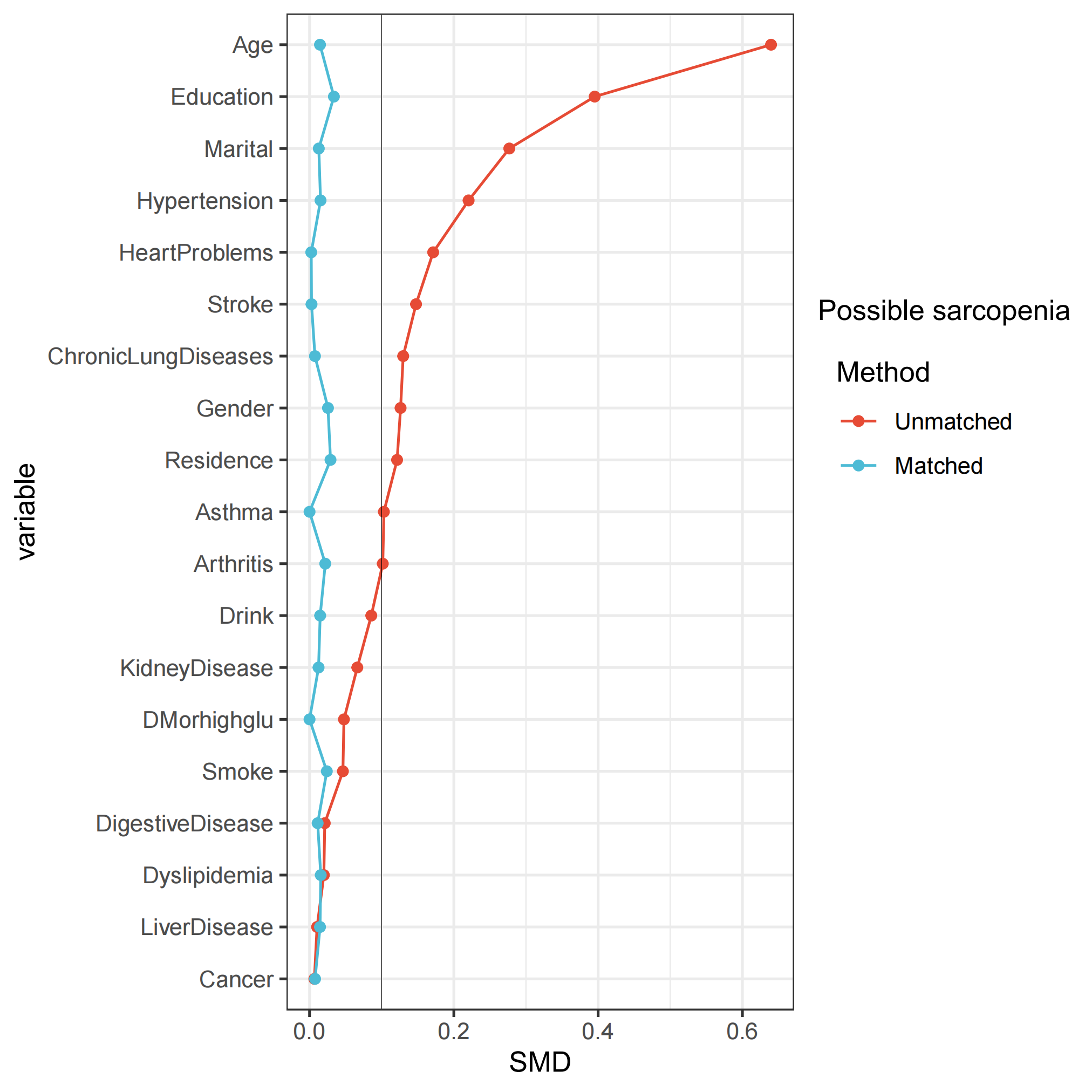
**

**Supplementary Figure 1. Standardized Mean Difference (SMD) for all covariates of probable sarcopenia**

**
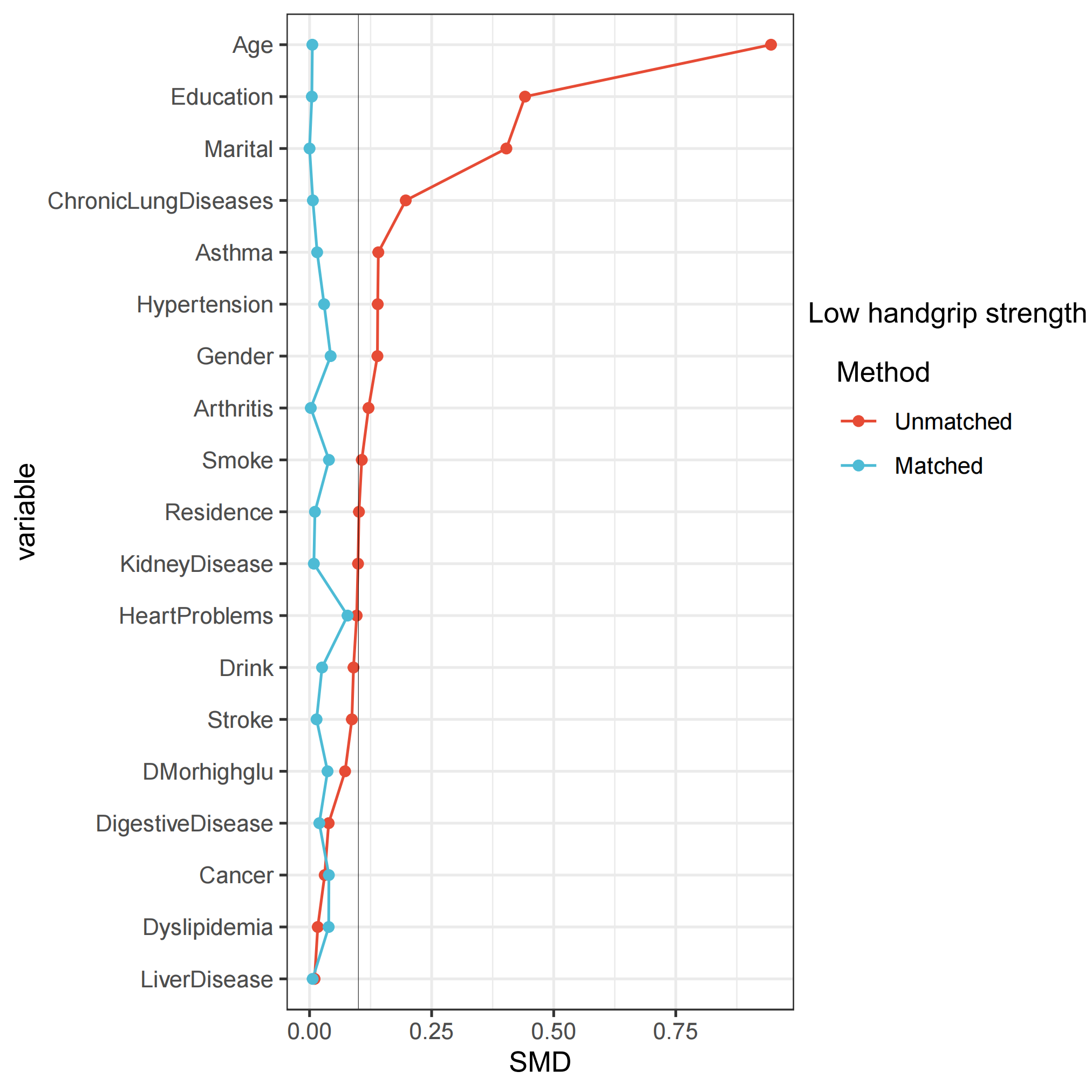
**

**Supplementary Figure 2. Standardized Mean Difference (SMD) for all covariates of low handgrip strength**

**
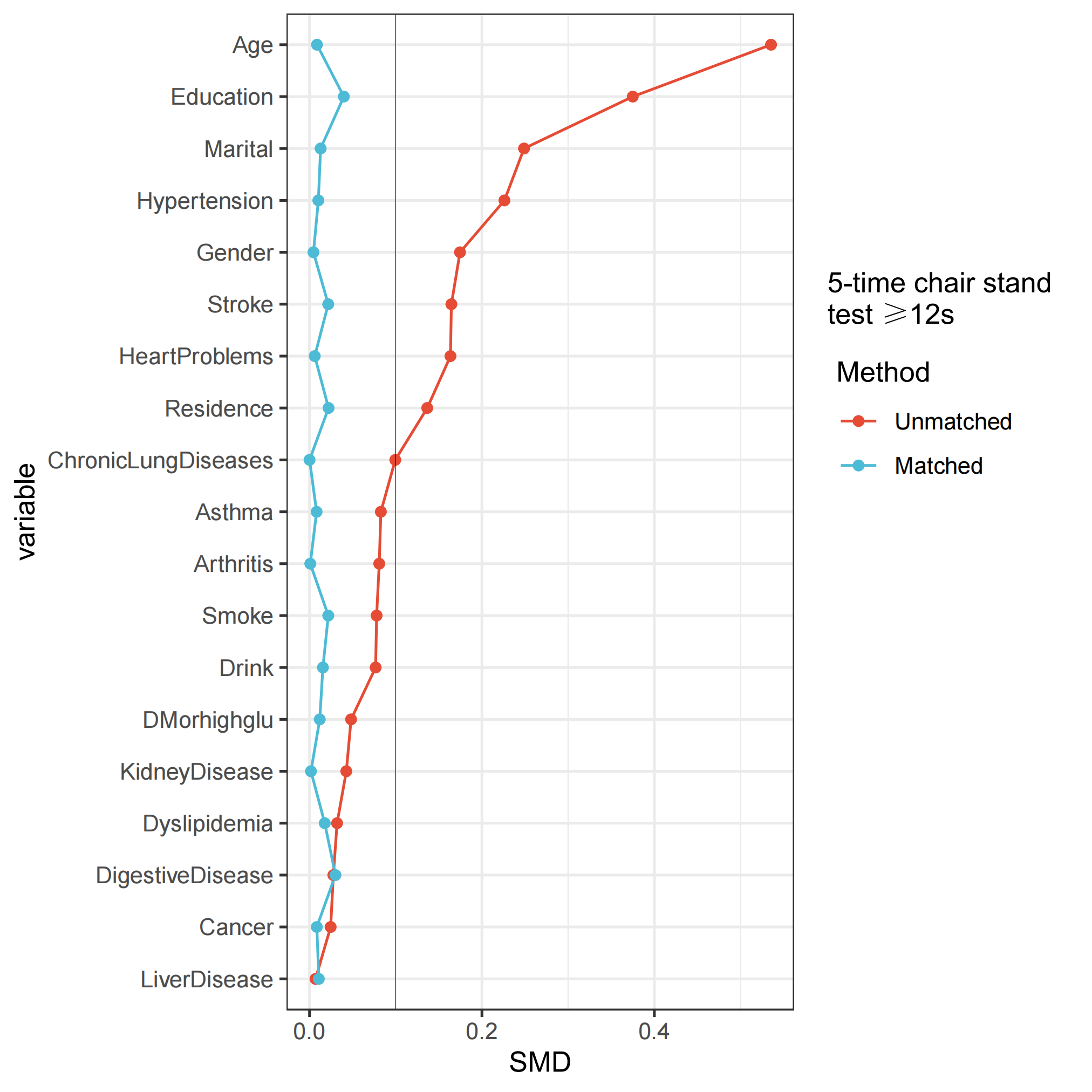
**

**Supplementary Figure 3. 5-time chair stand test ≥12s Standardized Mean Difference (SMD) for all covariates**
